# From stress to screen: family factors on the path to problematic media use in children aged 4–11

**DOI:** 10.3389/fpsyg.2025.1699094

**Published:** 2025-12-19

**Authors:** İbrahim Erdoğan Yayla, Samet Makas, Selami Yıldırım, Kübra Dombak, Eyüp Çelik

**Affiliations:** 1 Guidance and Psychological Counseling Department, Bayburt University, Bayburt, Türkiye; 2 Guidance and Psychological Counseling Department, Sakarya University, Sakarya, Türkiye; 3 Republic of Türkiye Ministry of National Education, Turkiye Cumhuriyeti Milli Egitim Bakanligi, Ankara, Türkiye

**Keywords:** parental stress, problematic media use, digital parenting awareness, family harmony, children

## Abstract

**Objective:**

Today, the rapid development of digital technologies fundamentally transforms how children interact with media. This research examines the mediating roles of parenting stress and problematic media use in children on the relationship between digital parenting awareness and family harmony.

**Methods:**

The data for this study were obtained from 502 parents with children aged 4–11 years. Participants’ daily screen time was 1–2 h (55.8%), 2–3 h (26.9%), 3–4 h (12%), 4+ h (5.4%). Convenience sampling was the method employed for the present study. Correlation analysis and structural equation modeling were performed to analyze the data.

**Results:**

The correlation analysis concluded that all variables were interrelated. Furthermore, the structural equation model demonstrated that parenting stress and problematic media use mediated the relationship between digital parenting awareness and family harmony.

**Conclusion:**

This study has contributed to understanding the relationship between parenting stress and problematic media use in childhood, as well as the variables that mediate this relationship. It has provided a new perspective on the variables that should be focused on in preventing and intervening in children’s problematic media use behaviors. Practically, the research results provide a valuable reference for university educators, assist school counselors in reducing students’ problematic media use behaviors through education, and help parents improve their skills in raising their digital awareness.

## 1 Introduction

Today, the rapid development of digital technologies is fundamentally transforming the way children interact with media. The widespread use of screen-based devices such as smartphones, tablets, laptops, and televisions has led to an increase in the amount of time children spend in front of screens (Hmidan et al., 2023; Salway et al., 2023). This situation has made digital media use not only a tool for accessing information and entertainment but also a factor that has a profound impact on children’s social, emotional, and cognitive development (Livingstone et al., 2015). Especially after the COVID-19 pandemic, the significant increase in the time children spend in online environments has also increased behavioral problems and screen addiction related to media use (Domoff et al., 2020; Eales et al., 2025). Problematic media use is defined as the excessive and uncontrolled use of screen-based media devices (e.g., computers, video games, smartphones, tablets, and televisions) that negatively affect a child’s social, behavioral, or academic functioning (Domoff et al., 2019). This concept focuses on the functional effects of media on children’s daily lives rather than the amount of screen time. Behavioral indicators such as an inability to reduce screen time, loss of interest in other activities, lying about media use, and excessive focus on media use are among the key indicators of problematic use (Domoff et al., 2019; Sigman, 2017). Unrestricted access to digital media tools increases the risk of children encountering age-inappropriate content, which can expose them to violence, sexuality, and other developmentally harmful content (Guerrero et al., 2019; Zoromba et al., 2023). Furthermore, increased digital media use at an early age can negatively impact the development of children’s communication skills and limit their opportunities to form face-to-face relationships with family members and peers (Poulain et al., 2018).

Research shows that approximately 10%–20% of young children exhibit symptoms of problematic media use (Barr et al., 2024). Various cross-sectional and longitudinal studies have found that excessive media exposure, particularly among children aged 0–10, is associated with psychosocial problems such as attention problems (Barton et al., 2021), emotional regulation difficulties (Wartberg et al., 2021), social withdrawal, and aggressive behavior (Zoromba et al., 2023). In a systematic review conducted by Rega et al. (2023), the findings of 35 studies focusing on children aged 5 months to 10 years were examined; it was found that older age groups exhibited more symptoms of problematic media use and that boys were at higher risk in this context. In another longitudinal study, it was noted that problematic media use symptoms increased over time in children aged 2.5–5.5 years; particularly, high screen use, emotional reactivity, and aggression in the early period played a decisive role in this development (Coyne et al., 2024).

On the other hand, a meta-analysis study revealing that problematic media use in children is shaped not only by individual but also by contextual factors found that parents’ attention divisions related to digital device use moderately increased the risk of problematic media use in children (Zhang et al., 2025). Additionally, factors such as parental burnout, depression, and stress were also found to increase children’s problematic media use (Derin et al., 2023; Pulkki-Råback et al., 2022). All these findings reveal that problematic media use in early childhood is a multidimensional problem shaped not only by individual characteristics but also by the interaction of parenting attitudes.

### 1.1 The Relationship between parental stress and problematic media use in children

Parenting plays a decisive role in child development, but it also brings with it various psychological and social responsibilities. The role expectations and daily demands parents face during this process can sometimes lead to stress and tension. Parental stress is defined as a state of psychological distress arising from the imbalance between the responsibilities assumed by the individual in their parental role and their capacity to meet these demands (Nielsen et al., 2020). When evaluated in the context of parenting, this definition shows that stress responses arise when the psychosocial demands faced by parents strain their own resources (Steele et al., 2020). Parental stress is a multidimensional construct shaped by the child’s needs, the parent’s perceived level of competence, environmental stress factors, and expectations regarding parental roles (Bao and Greder, 2023). The Parenting Stress Model suggests that stress can arise both in the parent domain (competence, attachment, isolation, etc.) and in the child domain (behavioral problems, compliance, attention problems, etc.) (Siracusano et al., 2021). In particular, challenging characteristics in children such as attention deficit, emotional regulation difficulties, and disruptive behaviors are strong predictors of increased parental stress levels (Molano et al., 2023). This situation can indirectly affect not only the parent’s mental health, but also the quality of care provided to the child, the form of interaction, and discipline strategies (Szymañska and Aranowska, 2021). Indeed, in situations where parental stress is high, digital media tools are used as a “digital sedative” to regulate children’s behavior, which can lead to a qualitatively problematic relationship between children and media (McDaniel and Radesky, 2020; Uzundağ et al., 2022).

Studies have shown that high parental stress weakens children’s ability to limit their media use and makes it difficult for parents to maintain consistent media mediation practices (Warren and Aloia, 2019; Wang et al., 2024). Barr et al. (2024) consider parenting stress to be one of the immediate environmental factors shaping children’s media use behaviors and argue that parental stress levels may increase the risk of problematic media use in children. Similarly, a study conducted with children aged 3–6 found a significant and positive relationship between parenting stress and problematic media use (Kattein et al., 2023). Shawcroft et al. (2024) have shown that postpartum stress and depression in mothers affect children’s long-term media usage habits. Uzundağ et al. (2022) reported that stress levels in mothers of children under the age of six predicted problematic media use in parents. This finding suggests that parental stress can influence problematic media use in children both directly and indirectly (e.g., through the parent’s own media habits, digital distraction, or decreased parental mediation). Modeling the time a child spends in a digital environment is another factor that reinforces problematic media use (Lauricella et al., 2015). At this point, it is thought that parental stress may have an impact not only on the individual level but also on family interaction processes. Parents with high stress levels may have low digital parenting awareness, which can undermine their ability to set boundaries, provide guidance, and serve as an appropriate model for their child’s digital media experiences (Akkaya et al., 2021). Furthermore, high stress can negatively impact family communication and emotional adjustment, posing an indirect risk factor for children’s digital behavior (Li et al., 2023).

Current research shows that digital parenting awareness is inversely related to problematic media use in children, meaning that children of parents with higher awareness have healthier media use habits (Barakat et al., 2025; Güzel and Öztürk, 2025). Similarly, children in families with higher family harmony and co-parenting have been found to exhibit more balanced digital media participation (Zhang and Song, 2025). Therefore, digital parenting awareness and family harmony can be considered mediating mechanisms in the relationship between parental stress and problematic media use in children.

### 1.2 The role of digital parenting awareness as a mediator in the relationship between parental stress and problematic media use in children

In the digital age, children’s relationship with media is shaped not only by individual preferences but also by parents’ digital attitudes, skills, and family interaction styles. According to Bronfenbrenner’s Ecological Systems Theory (1979), child development occurs through multilayered, interacting environmental systems. Within these systems, the family is at the center of the microsystem, and parents’ behaviors, attitudes, and psychological states directly shape children’s digital media experience. Therefore, parents’ digital awareness and stress levels can be considered important microsystem elements that determine the nature of children’s media use. In particular, high parenting stress weakens monitoring of children’s media use, content, and duration, which increases the risk of problematic media use in children (Wang et al., 2024). Indeed, a study found a significant negative relationship between parenting stress and digital parenting (Keleşoğlu and Adam, 2020). In this context, digital parenting awareness has emerged in recent years as both a protective factor and an explanatory variable in this relationship. Digital parenting awareness is defined as the parent’s ability to recognize the opportunities and risks of the digital world, to be aware of the effects of their own digital behavior on their child, and to consciously guide their child in the use of digital media (Yurdakul et al., 2013). Huang et al. (2018) address digital parenting awareness in four dimensions: digital safety, digital media literacy, parent-child digital communication, and role modeling awareness. These dimensions play a critical role in both protecting children from online risks and developing healthy digital habits.

A study conducted on children indicates that when digital parenting awareness is low, children’s media use becomes more problematic (Ereskici et al., 2024). In Arslan’s (2022) study, it was found that as the level of digital neglect among parents increased, the level of problematic media use among children aged 4–11 also increased accordingly. Similarly, Coşkunalp and Kuşcu (2022) reported that as parents’ digital competence decreases, the level of problematic media use among children aged 7–10 increases. In another study, it was reported that when parents have a high capacity to use digital tools consciously and guide their children, the time spent in the digital environment becomes more functional and media-related problems in children decrease (Yurdakul et al., 2013). In this regard, individuals with high digital parenting awareness can exhibit regulatory and guiding behaviors regarding their children’s media use, thereby preventing the development of problematic media use. In this context, it is thought that digital parenting awareness plays a mediating role in the relationship between parenting stress and problematic media use in children.

### 1.3 The mediating role of family harmony in the relationship between parental stress and problematic media use in children

In today’s rapidly digitizing world, children’s relationship with media is not limited to individual tendencies or technological capabilities; it is directly shaped by environmental factors such as family dynamics, parents’ mental health, and domestic relationships. In this context, parental stress emerges as one of the key factors negatively influencing the quality and quantity of time children spend in digital environments. Parents experiencing high levels of stress have weakened abilities to monitor, guide, and limit their children’s media use, which paves the way for problematic media use in children (Brauchli et al., 2024). Family harmony stands out as one of the main variables that can transform this negative process. Family harmony is defined as healthy communication, trust relationships, and emotional support networks established among family members (Olson, 2000). Strong family harmony facilitates effective parental guidance on media use and makes children’s time spent in the digital environment more functional (Coyne et al., 2014). In short, while parenting stress negatively affects children’s digital media use, strong family harmony balances these effects and plays a protective role in preventing problematic media use.

A study conducted on children reported that problematic media use symptoms significantly increased in cases where parent-child closeness was low (Swit et al., 2023). Additionally, a study conducted on early childhood revealed that children in families with low parent-child closeness are at increased risk of problematic media use not only in the current period but also in later years (Zhu et al., 2022). On the other hand, a study conducted in Shanghai during the COVID-19 restrictions found that psychological stress in mothers increased problematic media use in children; this effect was associated with mediating variables such as decreased mother-child interaction and family dysfunction (Wang et al., 2024). These findings indicate that problematic media use in children is closely related to the level of family harmony; when family interaction, emotional closeness, and structural functioning are weakened, problematic media use increases further. In this context, family harmony, i.e., the level of communication, cooperation, emotional closeness, and mutual support among family members, can be considered a critical factor that both regulates the effects of parenting stress and shapes child behavior.

Although high parental stress can sometimes lead to increased parental control, this control often occurs in an inconsistent, punitive, or reactive manner (Cenusã and Turliuc, 2024). Such inconsistent forms of control, rather than limiting children’s media use, increase parent-child conflict and may lead children to develop escape or covert use strategies (Yang et al., 2022). Similarly, increased control efforts under high stress may be counterproductive rather than reducing media use because stressed parents are often emotionally exhausted and unable to implement controlling behaviors sustainably (Chung et al., 2022). This suggests that despite high-stress parents’ increased control efforts, they cannot completely eliminate children’s opportunities to access media, and problematic media use may indirectly persist (Kattein et al., 2023). As a result, in environments with low family cohesion, excessive or inconsistent parental supervision due to stress may strengthen digital escape and addiction tendencies in children rather than creating a functional control mechanism. In this regard, it can be said that family harmony plays a mediating role in the relationship between parenting stress and problematic media use in children.

### 1.4 Present study

**FIGURE 1:**
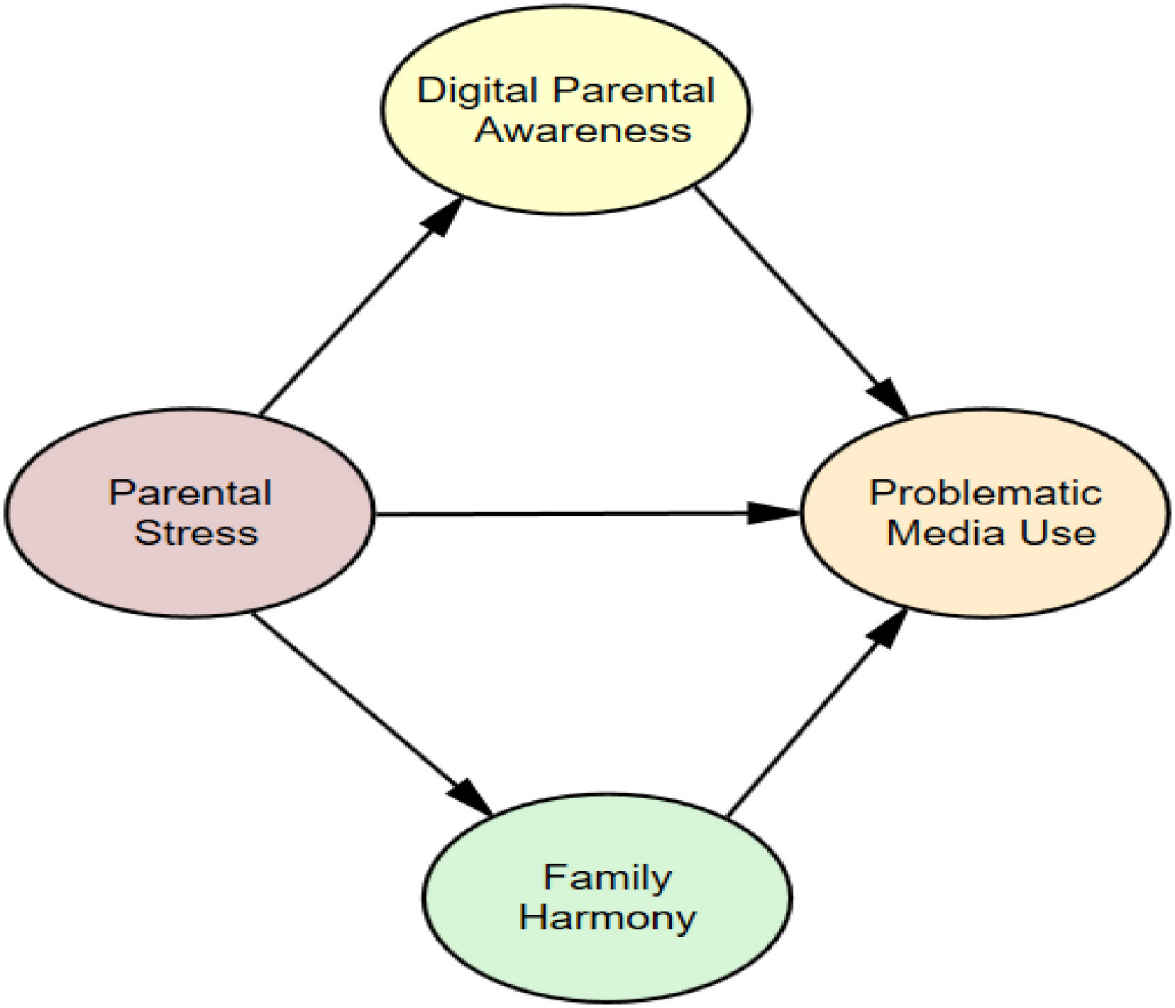
Hypothetical model.

The widespread use of digital media tools at an early age has brought with it new risk areas in children’s developmental processes. In particular, the increase in the amount of time children aged 4–11 spend with media can trigger a number of problematic situations, such as attention problems, social withdrawal, emotional regulation difficulties, and disruptions in family interactions. Problematic media use is particularly regarded as a developmental risk for children (Domoff et al., 2019). The literature provides strong evidence that parents’ psychosocial resources are decisive in this process, and that parental stress weakens their ability to regulate and limit their children’s interactions with digital media (Warren and Aloia, 2019; Wang et al., 2024). Given these negative effects, it has become important to investigate protective factors related to the family system and digital parenting. Recent studies have shown that digital parenting awareness is a multidimensional skill that encompasses parents’ awareness of their own media habits, their awareness of modeling for their children, and their ability to develop protective media guidance against online risks (Huang et al., 2018). This high level of awareness can reduce the impact of parenting stress on children and bring children’s media use within healthier limits. On the other hand, family harmony also emerges as an important factor that can transform this relationship. Emotional closeness, open communication, and supportive relationships within the family can play a balancing role in the relationship that a stressed parent establishes with their child in the context of digital media (Coyne et al., 2014; Olson, 2000). Accordingly, the current study aims to examine the mediating roles of digital parenting awareness and family cohesion in the relationship between parenting stress and problematic media use in children. This study makes a unique contribution to the literature as one of the first to incorporate both mediating variables within a single structural model. By integrating the domains of family functioning and digital parenting, the study aims to provide a theoretical basis for early intervention and preventive strategies to prevent problematic media use in children. Consequently, the following hypotheses (as seen Figure 1) were tested in this study.

H1: There is a positive correlation between parental stress and problematic media use in children.

H2: Digital parenting awareness and family harmony play a mediating role in the relationship between parenting stress and problematic media use in children.

H3: Digital parenting awareness plays a mediating role in the relationship between parenting stress and family harmony.

H4: Family harmony plays a mediating role in the relationship between digital parenting awareness and problematic media use in children.

## 2 Materials and methods

### 2.1 Participants

The data for this study were obtained from 502 parents with children aged 4–11 years. 470 (93.6%) mothers and 32 (6.4%) fathers participated in the study, with a mean age of 36.63 years (SD = 5.25, range = 26–53). Participants’ daily screen time was 1–2 h (55.8%), 2–3 h (26.9%), 3–4 h (12%), 4+ h (5.4%). Convenience sampling was the method employed for the present study.

### 2.2 Instruments

#### 2.2.1 Digital Parental Awareness Scale

The scale was developed by Manap and Durmuş (2020) to assess the awareness levels of parents regarding their roles in digital environments. The questionnaire consists of 16 items and a 5-point Likert scale. The scale is composed of four subscales: Protecting from risks, efficient usage, digital negligence and being a role model. The Cronbach’s alpha coefficient ranges from 0.634 to 0.799 for the subscales. The DFA results (χ^2^/df = 1.32; SRMR = 0.06; AGFI = 0.93; RMSEA = 0.03; CFI = 0.98; NFI = 0.91; GFI = 0.95) indicate good fit indices.

#### 2.2.2 Problematic Media Use Scale: a parent report measure of screen addiction in children

The scale measures problematic use of visual media devices (TV, tablet, computer, smartphone, etc.) by children, as perceived by parents. It consists of 9 items, is a 5-point Likert scale, and is unidimensional; a higher score indicates more problematic use. The original version was developed by Domoff et al. (2019), and the Turkish version was adapted by Furuncu and Öztürk (2020). The Turkish adaptation was prepared through two independent forward translations, blind back translations, and expert panel consensus; it was implemented after a brief cognitive control/pilot study. In this study, CFA showed good fit (χ^2^/df = 1.81; IFI = 0.98; GFI = 0.91; RMSEA = 0.06; CFI = 0.99; SRMR = 0.04) and high internal consistency (Cronbach’s α = 0.90).

#### 2.2.3 Family Harmony Scale

The Family Cohesion Scale–Short Form was developed to assess individuals’ perceptions of family cohesion. The original scale was developed by Kavikondala et al. (2016). The Turkish adaptation by Kula et al. (2018) was carried out through a multi-stage translation process: preliminary translations were obtained from multiple experts, conceptual equivalence was ensured through expert panel evaluation with specialists in the field of Educational Sciences, and then linguistic consistency was verified through independent back-translation, and the text was finalized. It consists of 5 items and a 5-point Likert scale. The scale is unidimensional, and the higher the score, the higher the level of adjustment. The adaptation study supported the single-factor structure through confirmatory factor analysis [χ^2^_(5, *N* = 483)_ = 17,739, *p* < 0.05; RMSEA = 0.073; CFI = 0.99; SRMR = 0.016; TLI = 0.99; item loadings 0.70–0.89]. Internal consistency is high (Cronbach’s α = 0.91), and within the scope of criterion-related validity, the scale showed positive relationships with life satisfaction and subjective wellbeing.

#### 2.2.4 Parental Stress Scale

The Parental Stress Scale was used to assess the stress levels experienced by parents [Original: Berry and Jones (1995); Turkish adaptation: Gördesli and Aydın Sünbül, 2021]. The English version of the scale was translated into Turkish by the authors and five PhD-level academics specializing in the field; the appropriateness of the items was examined using a 5-point Likert-type evaluation form, and the scale was finalized in line with the opinions of Turkish language and measurement-evaluation experts. The scale is a 5-point Likert scale consisting of 16 items and four sub-dimensions: parental rewards, parental stress factors, lack of control, and parental satisfaction. Confirmatory factor analysis (CFA) results indicate that the model fits well (χ^2^/df = 1.56, SRMR = 0.05, CFI = 0.96, GFI = 0.94, RMSEA = 0.04, AGFI = 0.93). The Cronbach’s alpha coefficient ranges from 0.70 to 0.86 for the subscales and is 0.81 for the total scale.

### 2.3 Procedure

This study was conducted in June-July 2025 with parents of students from 15 different kindergartens and primary schools in three different cities in Turkey. Parents were invited to participate by sharing an online form link via school-parent communication groups. The announcement stated that participation was voluntary, provided information about privacy and scientific use principles, and indicated the informed consent section prior to the survey. The study was conducted after obtaining the necessary permissions from Bayburt University Scientific Research and Ethics Committee (Date: 27 June 2025; Approval No: 357).

### 2.4 Data analysis

To validate our hypotheses, we followed a three-step process. First, before statistical analysis, we calculated Pearson correlations and descriptive statistics between all variables using SPSS 27.0. Second, we created a measurement model using AMOS 24 Graphics software to identify the relationships between parental stress, digital parental awareness, family harmony, and problematic media use. Finally, a structural equation model was established based on the measurement model and theory and tested using AMOS 24 Graphics software.

## 3 Results

### 3.1 Preliminary analyses

Table 1 presents the correlations between problematic media use, family harmony, parental stress sub-dimensions (parental rewards, parental stressors, lack of control and parental satisfaction) and digital parental awareness sub-dimensions (protecting from risks, efficient usage, digital negligence and being a role model).

**TABLE 1:** Findings regarding the relationships between variables and descriptive statistic.

Variables	1	2	3	4	5	6	7	8	9	10
1. Problematic media use	1									
2. Parental rewards	0.30**	1								
3. Parental stressors	0.24**	0.35**	1							
4. Lack of control	0.22**	0.56**	0.53**	1						
5. Parental satisfaction	0.25**	0.64**	0.40**	0.59**	1					
6. Family harmony	−0.57**	−0.30**	−0.17**	−0.19**	−0.28**	1				
7. Protecting from risks	−0.41**	−0.30**	−0.14**	−0.21**	−0.24**	0.24**	1			
8. Efficient usage	−0.35**	−0.29**	−0.17**	−0.19**	−0.25**	0.22**	0.87**	1		
9. Digital negligence	−0.57**	−0.25**	−0.25**	−0.24**	−0.24**	0.29**	0.46**	0.43**		
10. Being a role model	−0.42**	−0.21**	−0.27**	−0.21**	−0.22**	0.25**	0.46**	0.44**	0.56**	1
Mean	19.21	7.38	12.86	4.58	3.21	18.40	16.28	16.21	15.97	16.94
SD	7.38	2.58	4.44	1.92	1.23	5.46	2.78	2.59	2.90	2.61
Skewness	0.77	1.17	0.37	1.44	1.02	−0.59	−0.85	−0.85	−0.88	−1.11
Kurtosis	−0.15	1.74	−0.46	1.73	1.25	−0.87	0.31	0.63	0.50	1.02

**p < 0.01.

When Table 1 is examined, it can be seen that the skewness values range from −1.11 to 1.44, while the kurtosis values range from −0.87 to 1.74. These values are between +2 and −2, which is within the acceptable range for normality (Tabachnick and Fidell, 2015).

Before proceeding to the structural equation model analysis, multicollinearity among the variables was examined. For this purpose, the variance inflation factor (VIF), Tolerance, and Condition Index values were evaluated. As shown in Table 2, the VIF values ranged from 1.148 to 1.209, the Tolerance values ranged from 0.827 to 0.871, and the Condition Index values were below 30. These results indicate that there is no multicollinearity problem in the model.

**TABLE 2:** Results of multicollinearity analysis among variables.

Variables	Condition Index	VIF	Tolerance
1. Problematic media use	1	–	–
2. Parental stress	6,125	1,185	0.844
3. Digital parental awareness	10,602	1,148	0.871
4. Family harmony	25,115	1,209	0.827

### 3.2 Measurement model

**FIGURE 2:**
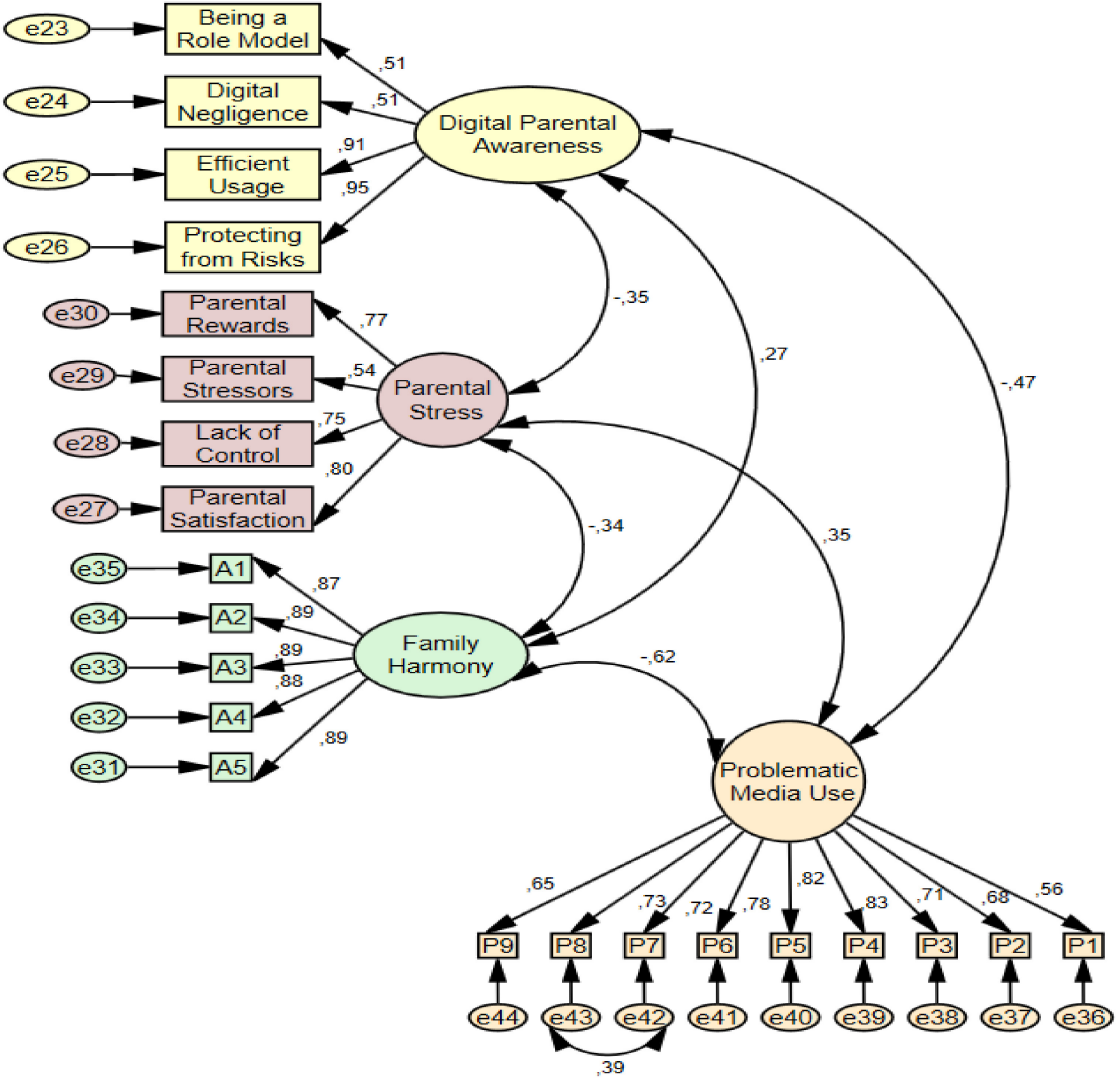
The covariances between digital parental awareness, parental stress, family harmony and problematic media use.

**FIGURE 3:**
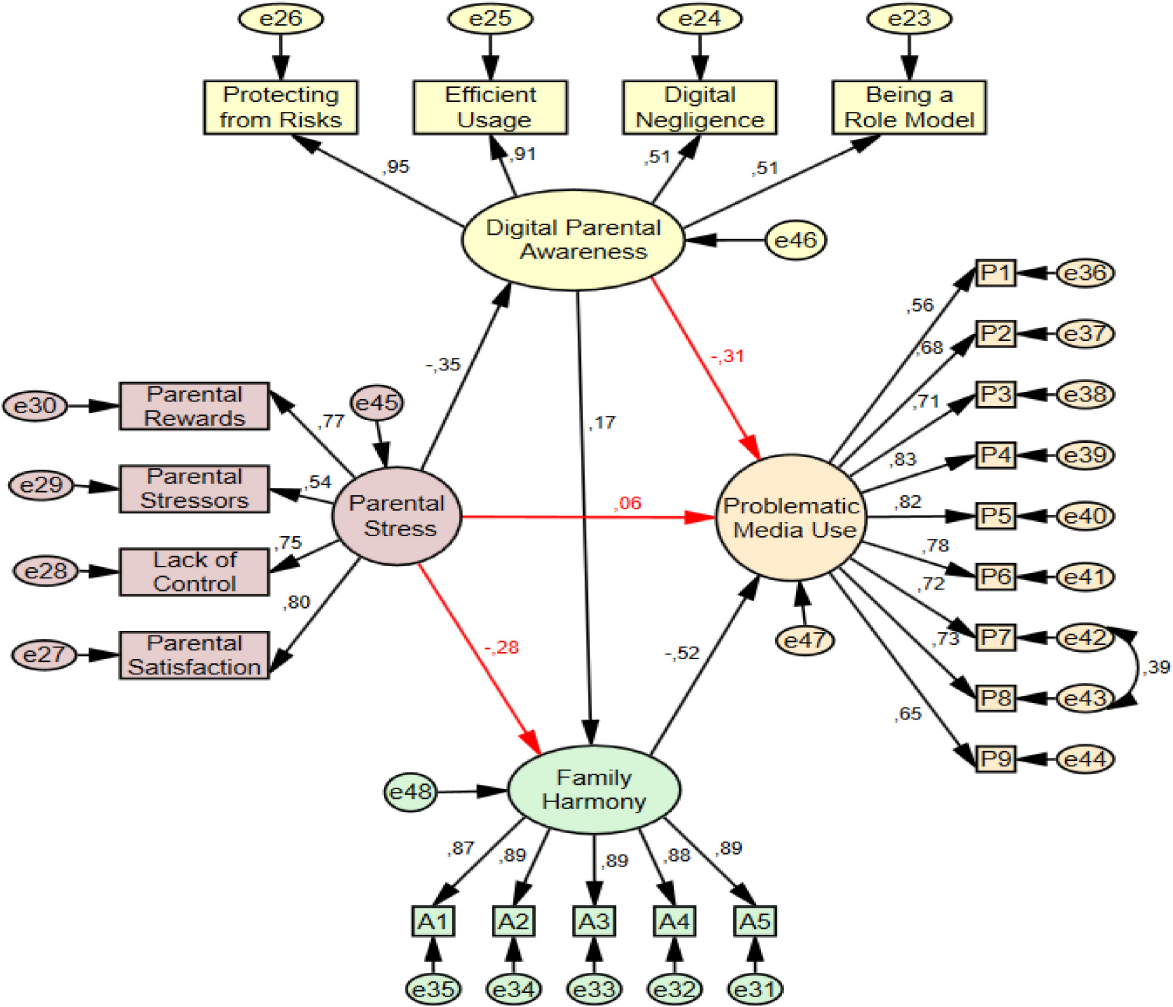
The result of mediation model.

In the study, before testing the structural equation model for the mediating roles of digital parental awareness and family harmony between parental stress and problematic media use, a measurement model was created to determine the consistency between the variables. The measurement model is presented in Figure 2 and the structural equation model is presented in Figure 3.

When examining the model in Figure 2, it was found that the covariance between parental stress and problematic media use (cov = 0.35, *p* < 0.001), parental stress and digital parental awareness (cov = −0.35, *p* < 0.001), digital parental awareness and problematic media use (cov = −0.47, *p* < 0.001), family harmony and digital parental awareness (cov = 0.27, *p* < 0.001), parental stress and family harmony (cov = −0.34, *p* < 0.001), family harmony and problematic media use (cov = −0.62, *p* < 0.001) were statistically significant. Moreover, the fit indices for the measurement model in Figure 2 are acceptable (χ^2^/df = 4.05, SRMR = 0.08, GFI = 0.86, IFI = 0.92, CFI = 0.91, RMSEA = 0.08).

### 3.3 The result of mediation model

The findings regarding the mediating role of digital parental awareness and family harmony in the relationship between parental stress and problematic media use are presented in Figure 3.

Through structural equation modeling, the indirect effects of digital parental awareness and family harmony on parental stress and problematic media use were examined. The structural model created in line with the findings obtained from the measurement model and the literature is presented in Figure 3. When examining Figure 3, the relationship between parental stress and digital parental awareness (β = −0.35, *p* < 0.001), the relationship between digital parental awareness and problematic media use (β = −0.31, *p* < 0.001), the relationship between parental stress and family harmony (β = −0.28, *p* < 0.001), the relationship between family harmony and problematic media use (β = −0.52, *p* < 0.001), the relationship between digital parental awareness and family harmony (β = 0.17, *p* < 0.001), and the relationship between parental stress and problematic media use (β = −0.06, *p* > 0.001) were found. In order to evaluate the appropriateness of the model, the acceptable fit index ranges suggested in the literature (Hooper et al., 2008; Hu and Bentler, 1998; Marsh and Hau, 1996; Wheaton et al., 1977) were taken into consideration and the fit indices obtained for the tested mediation model are reported in Table 3.

**TABLE 3:** Acceptance ranges for fit indices and fit indices obtained from the mediator model test.

Indices	Perfect fit limit	Acceptable fit limit	Measurement model indices	Mediator model indices	Result
X^2^/SF	0–2.5	≤ 5	4.05	4.05	Acceptable
RMSEA	≤ 05	≤ 08	0.08	0.08	Acceptable
CFI	≥ 95	≥ 90	0.91	0.91	Acceptable
GFI	≥ 90	≥ 85	0.86	0.86	Acceptable
SRMR	≤ 05	≤ 08	0.08	0.08	Acceptable
IFI	≥ 95	≥ 90	0.92	0.92	Acceptable

When the measurement model in Figure 2 and the structural equation model in Figure 3 were analyzed together, it was seen that digital parental awareness and family harmony played a full mediating role between parental stress and problematic media use (c = 0.35, *p* < 0.001; c′ = 0.06, *p* > 0.001). In addition, family harmony played a partial mediation role between digital parental awareness and problematic media use (c = −0.47, *p* < 0.001; c′ = −0.31, *p* < 0.001). Digital parental awareness showed a partial mediation role between parental stress and family harmony (c = −0.34, *p* < 0.001; c′ = −0.28, *p* < 0.001). The significance of direct, indirect, and total effects in mediation analyses was assessed using the bootstrap method on a sample of 5,000. The fact that the obtained 95% bias-corrected confidence intervals did not include the zero value indicates that the relevant indirect effects are statistically significant (as seen Table 4).

**TABLE 4:** Standardized path coefficients and bootstrap results for the mediation model.

Path	Coefficient (β)	SE	95% CI	
Standardized total effect			Lower	Upper
Parental stress–digital parental awareness–family harmony	−0.34	0.04	−0.432	−0.258
Digital parental awareness–family harmony–problematic media use	−0.40	0.05	−0.491	−0.288
Parental stress–digital parental awareness–family harmony–problematic media use	0.35	0.06	0.238	0.455
**Standardized direct effect**				
Parental stress–digital parental awareness–family harmony	−0.28	0.05	−0.385	−0.183
Digital parental awareness–family harmony–problematic media use	−0.31	0.05	−0.394	−0.217
Parental stress–digital parental awareness–family harmony–problematic media use	0.06	0.05	−0.047	0.162
**Standardized indirect effect**				
Parental stress–digital parental awareness–family harmony	−0.06	0.02	−105	−0.026
Digital parental awareness–family harmony–problematic media use	−0.09	0.02	−0.147	−0.038
Parental stress–digital parental awareness–family harmony–problematic media use	0.29	0.03	0.227	0.354

## 4 Discussion

In this study, the mediating roles of digital parenting awareness and family harmony in the relationship between parenting stress and problematic media use in children were examined. The research findings revealed that digital parenting awareness and family harmony played a full mediating role in the relationship between parenting stress and problematic media use in children. A significant relationship was found between parental stress and problematic media use, and this relationship was found to be mediated by digital parenting awareness and family harmony. This finding suggests that the effect of parental stress on children’s problematic media use is mediated by family factors such as parenting awareness and family harmony. When reviewing the literature, it is observed that there are numerous research findings supporting this study’s results. The results of the study conducted by Wang et al. (2024) reveal that there is a direct relationship between parental stress and children’s media usage behaviors, and that this relationship is also indirectly strengthened through parents’ inappropriate attitudes on digital platforms, i.e., digital parenting awareness. Research conducted by Nielsen et al. (2019) revealed a significant relationship between parents’ levels of digital awareness and adolescents’ problematic media use behaviors; as family harmony decreases, parents experience difficulties in guiding their children’s media use behaviors, and this situation increases children’s risky behaviors in the digital environment. Similarly, a study by Geng et al. (2023) observed a positive correlation between children’s electronic media use and internalization difficulties. Family cohesion played a mediating role in this relationship. All these research results support the basic components of the structural model discussed in this study. When looking at the literature, it is observed that high stress levels in parents pose a significant risk to children’s cognitive, social, and emotional development (Crnic and Low, 2002; Östberg and Hagekull, 2000). This stress is seen as an important risk factor for problematic media use among children, especially in today’s technology-intensive world. This is because when parental stress is high, parents use digital media tools as a behavior regulation or behavior control tool to regulate their children’s behavior, and the repeated use of this method can lead to problematic relationships between children and media (McDaniel and Radesky, 2020; Uzundağ et al., 2022). Many studies have also yielded results that support this view, revealing a significant and positive relationship between parenting stress and problematic media use (Kattein et al., 2023; Uzundağ et al., 2022; Warren and Aloia, 2019). However, increased awareness of digital parenting has played a balancing role in preventing parenting stress from leading to problematic media use among children. This is because parents with increased digital parenting awareness have been observed to gain the ability to recognize the risks of the digital world, understand the effects of their own digital behavior on their children, and consciously guide their children in the use of digital media (Yurdakul et al., 2013). Furthermore, the increase in the level of digital parenting has been an important variable in reducing children’s problematic media use by increasing family harmony, thereby increasing interaction and sharing among family members and providing opportunities for quality time within the family, thus preventing unconscious media use (Aydın and Ataş, 2025). Based on all this literature, it is possible to say that the negative role of parental stress on children’s problematic media use behavior can be significantly mitigated through variables such as digital parenting awareness and family harmony.

Another important finding from this study is that digital parenting awareness plays a partial mediating role in the relationship between parenting stress and family harmony. In other words, while family harmony decreases as stress levels increase in parents, the negative effect caused by stress is balanced to a certain extent by digital parenting awareness. When examining the literature, it is evident that with the advent of the digital age, parents’ responsibilities in raising children have increased and become more complex. This situation has also led to an increase in parental stress. This increased stress negatively affects family harmony (Connell and Strambler, 2021; Chung et al., 2022; Griffith, 2020). However, this study observed that the relationship in question is not limited to the effect of these two variables on each other, but also takes shape at different levels depending on the level of digital parenting awareness. Individuals with increased parenting stress sometimes need secondary resources to reduce their parenting demands. Digital media tools are highly suitable instruments for this purpose due to their inherent characteristics. Parents try to cope with their stress by allowing their children to be occupied with these activities, thereby reducing their parenting demands (Pempek and McDaniel, 2016; Warren and Aloia, 2019). Sometimes, individuals are observed to use digital media tools directly to cope with their stress and achieve momentary relief (Yıldırım, 2021). Although this method is preferred as an immediate solution for parents, it can eventually turn into problematic behavior and may also affect family relationships. As a result, the quality time spent within the family decreases, and family harmony is harmed by this process. As family sharing decreases, family members become more individualistic within the family and create their own time dynamics. This situation poses social, emotional, and cognitive risks for family members (Ardıç and Selvi, 2022). This poses an even greater risk for children of play age within the family. In today’s technological age, it has been observed that children in families where sharing and quality time are insufficient use technological devices as a means of coping with feelings of loneliness during their free time (Deniz and Kazu, 2021). Over time, this situation can turn into a problem such as behavioral addiction, causing increased conflict between parents and children and negatively affecting family harmony. Therefore, individuals experiencing parenting stress can partially control parenting stress and maintain family harmony by increasing their digital parenting awareness and improving their parenting competence in today’s world, where parenting roles are being redefined (Livingstone and Helsper, 2008; Nikken and Schols, 2015). In conclusion, these findings reveal that digital parenting awareness is not merely a competence aimed at managing children’s media usage behavior, but also a protective factor that regulates parental stress responses and supports family harmony.

Finally, this study examined the mediating role of family harmony in the relationship between digital parenting awareness and problematic media use in children; it was concluded that family harmony plays a partial mediating role between digital parenting awareness and problematic media use. This result shows us that children of parents with high levels of digital parenting awareness exhibit less problematic media use behavior, and that this effect is partially explained by family harmony. When reviewing the literature, it is observed that there are no research results supporting this research finding. However, a review of the literature reveals that the higher the digital parental awareness, the more protective and supportive the approach (Akkaya et al., 2021). Furthermore, a positive relationship was found between digital parental awareness and the parent-child relationship (Toran et al., 2024). On the other hand, a negative relationship has been observed between family harmony and social media use (Ercengiz and Bayraktar, 2025; Swit et al., 2023; Zhu et al., 2022; Wang et al., 2024) and a positive relationship between family conflict and problematic social media use (Yıldırım et al., 2025). Similarly, a study conducted by Manap and Durmuş (2021) found that parents with healthy communication and harmony within the family had lower levels of negative modeling and digital neglect compared to parents with low levels of family harmony, and higher levels of effective use of digital media tools and protection from risks. Finally, it is stated that there is a positive relationship between positive family processes and adolescents’ self-concept, self-control, and youth perspective-taking (Yeung, 2021), and that parental supervision and active communication are effective in reducing young people’s problem behaviors (Lippold et al., 2013). All these research results show that family harmony is an effective factor in children’s media usage behavior and that digital parenting awareness plays an important bridging role in the relationship between media behavior. This is because situations such as parents being unable to communicate with their children due to the use of technological devices or focusing on digital tools while ignoring family members present can damage family harmony and lead to problematic media use in children.

## 5 Conclusion

In this study based on structural equation modeling, the mediating roles of digital parenting awareness and family harmony in the relationship between parenting stress and problematic media use in children were examined, and important findings were obtained. First, the relationships between the variables were analyzed; it was found that the relationship between parental stress and problematic media use was positively significant, the relationship between digital parenting awareness and problematic media use was negatively significant, and the relationship between parental stress and family harmony was negatively significant. Subsequently, the findings from the study revealed that digital parenting awareness and family harmony variables played a full mediating role in the relationship between parental stress and children’s problematic media use. Another important finding from this study is that digital parenting awareness plays a partial mediating role in the relationship between parental stress and family harmony. In other words, while family harmony decreases as stress levels increase in parents, the negative effect caused by stress is balanced to a certain extent by digital parenting awareness. Finally, this study examined the mediating role of family harmony in the relationship between digital parenting awareness and problematic media use in children; it was concluded that family harmony has a partial mediating role between digital parenting awareness and problematic media use.

Practically, the research results can provide a useful reference for researchers, assist in addressing children’s problematic media use behaviors and improving family harmony, and optimize education.

### 5.1 Contribution and limitations

Theoretically, this study has contributed to understanding the relationship between parenting stress and problematic media use in childhood, as well as the variables that mediate this relationship. It has provided a new perspective on the variables that should be focused on in terms of preventing and intervening in children’s problematic media use behaviors. Practically, the research results can provide a useful reference for university educators, assist school counselors in reducing students’ problematic media use behaviors through training, and help parents improve their skills in raising their digital awareness.

First, the sample size of this study is relatively small, and based on this, future studies should strive to increase the sample size and expand the scope of the study to more regions and different types of schools in order to increase the representativeness and reliability of the study. Additionally, because 93.6% of the study participants were mothers, this creates a limitation in generalizing the research results to fathers. However, the reason for this situation is that mothers mostly participate in studies on children studying in Türkiye. Another limitation of the study is that the results cannot be generalized to populations in other cultures, as they are limited to Turkish culture. Future research could compare problematic media use across different age groups, evaluate the effectiveness of various strategies to prevent problematic media use, and investigate the impact of time spent at school on students’ mental health. Experimental studies can be used to assess the effects of parents’ digital awareness on children’s problematic media use behaviors. Meta-analytic studies will be valuable for conducting a comprehensive review of the existing literature in this field, synthesizing the results of various studies, and ultimately deriving standard conclusions from research findings. Mixed-method studies combining both quantitative data and in-depth qualitative analysis can provide a more comprehensive understanding of parental stress and problematic media use and facilitate the comparison of the data obtained. In conclusion, the findings from this study lay the groundwork for future research in this area and provide valuable insights that can strengthen subsequent experimental and meta-analytic studies, thereby guiding new research in this field.

## Data Availability

The raw data supporting the conclusions of this article will be made available by the authors, without undue reservation.
